# Advancing Home Rehabilitation: The PlanAID Robot’s Approach to Upper-Body Exercise Through Impedance Control

**DOI:** 10.3390/s26010175

**Published:** 2025-12-26

**Authors:** David Breton, Thierry Laliberté, Andréanne K. Blanchette, Alexandre Campeau-Lecours

**Affiliations:** 1 Centre Interdisciplinaire de Recherche en Réadaptation et Intégration Sociale (Cirris), Quebec City, QC G1M 2S8, Canadaalexandre.campeau-lecours@gmc.ulaval.ca (A.C.-L.); 2 Robotics Laboratory, Department of Mechanical Engineering, Université Laval, Quebec City, QC G1V 0A6, Canada; 3 School of Rehabilitation Sciences, Faculty of Medicine, Université Laval, Quebec City, QC G1V 0A6, Canada

**Keywords:** rehabilitation engineering, rehabilitation robotics, motor learning, impedance control, control engineering, force sensing

## Abstract

Rehabilitation robots are a leading solution towards bridging the gap between the growing number of rehabilitation patients requiring therapy and the limited availability of healthcare professionals. However, existing robotic systems are often bulky and expensive, limiting their ability to provide widespread, repetitive, and intensive exercises. This paper presents the development of an impedance-based control strategy designed to provide safe and compliant upper-body passive and active exercises on the low-cost PlanAID robot, which is built using consumer-grade components. The system’s functionalities are evaluated using a high-precision force sensor. Results show that the PlanAID exhibits performance comparable to seminal devices such as the MIT-Manus, achieving a similar applicable reaction force target of 28 N and reflected inertia of 1.1 kg. Although the overall performance is comparable, the low-cost PlanAID prototype suffers from reduced coupled stability margins, limiting the maximum achievable virtual spring constant to 1100 N/m. Despite this limitation, the stiffness values required in practical applications remain low, suggesting that the PlanAID could potentially be a viable candidate for real-world rehabilitation. Initial user feedback was obtained through a preliminary qualitative trial involving healthy subjects.

## 1. Introduction

The prevalence of neurological conditions is rising, primarily due to population aging and increased life expectancy after a lesion. Although the number of healthcare professionals is increasing, there is still a gap between the supply of services and the needs of the population to be met [1]. This imbalance makes conventional therapy methods increasingly labour-intensive and time-limited per patient, which can negatively impact recovery. However, early, repetitive, and intensive rehabilitation exercises are often critical in achieving optimal recovery outcomes [2,3]. The use of rehabilitation robots may represent a viable strategy to address this issue by enhancing the rehabilitation process. Indeed, such technologies enable individuals with upper-limb sensorimotor impairments to perform a higher number of exercise repetitions and to carry out a greater range of daily activities with increased autonomy.

Rehabilitation robots have been in development since the early 1990s, with the MIT Manus—now known commercially as InMotion—being one of the earliest examples. This end-effector robot, with two planar degrees of freedom (DoF), set the foundation for future advancements in robotic rehabilitation [4]. Over the years, various designs have been explored, including serial 3-DoF robots and exoskeleton robots [5]. Despite these innovations, the planar end-effector design remains widely used in contemporary systems, such as the REAplan [6] and Kinarm [7], due to its proven efficacy in clinical trials [8] and cost-effectiveness compared to 3-DoF robots. Nevertheless, most commercial rehabilitation robots remain bulky and expensive, with prices ranging from CAD 50,000 to CAD 250,000, limiting their widespread use to research and specialized rehabilitation centres. The proposed innovative solutions, such as high-torque direct-drive motors and precision sensors, reflect a clear commitment to optimizing the rehabilitation of individuals with upper-limb sensorimotor impairments. While these features optimize performance, they also contribute to increased costs and bulk.

To make technologies more accessible, alternatives that are low-cost and easy to use are needed, allowing larger-scale adoption while still enabling adequate repetitiveness and intensity for effective exercising. In response to this demand, the PlanAID robot was developed [9]. Cost reduction was achieved by leveraging consumer-grade electronics and opting for an actuation design similar to the MIT mini cheetah, which is known for its high torque and transparency [10], as well as a low actuator cost [11].

The PlanAID robot is designed for and aims to support both passive and active rehabilitation exercises, enabling limb movement either with external assistance or through the user’s own effort. Passive mobilization exercises help to maintain or improve joint mobility, as well as to prevent certain complications secondary to the lesion, such as contractures [12,13]. The movements are therefore performed by an external source, without any voluntary muscle contraction from the individual. Passive exercises are particularly used in the early stages of recovery. Active exercises are introduced into rehabilitation as soon as the person is capable of contributing to the movement. These exercises aim to maintain or restore sensorimotor function [14]. Passive exercises on the PlanAID are implemented using a guided trajectory, while an active trajectory is implemented using a tunnelling strategy using virtual walls.

While passive exercises can be effectively managed using conventional robot position control methods [13], active exercises require a dynamic interaction between the robot and the user. As such, compliant rehabilitation exercise control is programmed using an impedance control strategy—widely recognized as a preferred approach for human–robot interaction [13]. Impedance control is made possible thanks to the PlanAID’s designed backdrivability and low inertia, which allow it to respond naturally to user-initiated movements. Additionally, a custom-built multi-axis force sensor provides real-time force feedback, enabling accurate friction compensation and enhancing the transparency of interaction.

This paper presents the development and experimental validation of a control strategy implemented on an improved PlanAID prototype, designed to provide both passive and active exercises using an impedance control scheme and low-cost electronics. The experimental evaluation aims to validate the robot’s functionality and control approach, serving as a preliminary step toward future improvements and clinical trials. The Section 2 describes the prototype’s key features, including actuation and sensing components, as well as the robot’s kinematics and control strategy. The experimental validation methodology and associated objectives are also detailed. The Section 3 presents the experimental validation outcomes along with a qualitative assessment of users’ perceptions of the device. Finally, the paper concludes with a summary of the findings and perspectives for future work.

## 2. Materials and Methods

### 2.1. Mechanical Design

**Figure 1:**
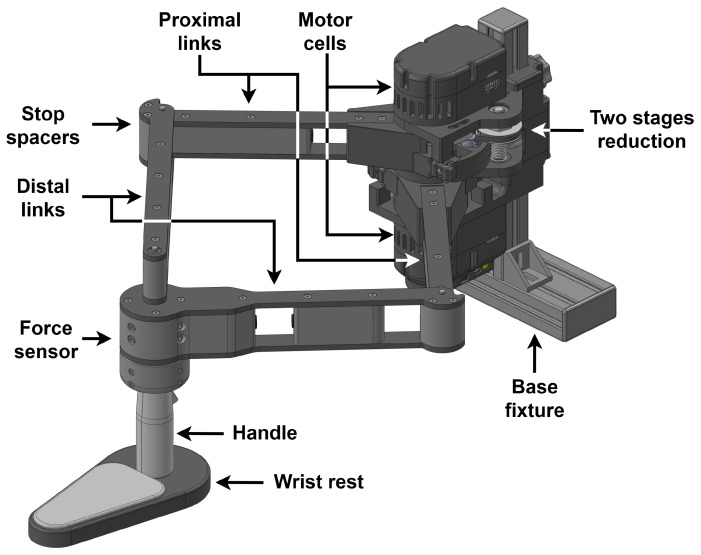
Computer-aided design (CAD) model of the PlanAID robot.

The robot, shown in Figure 1, is a 2-DoF five-bar linkage parallel robot with a symmetrical design, meaning that both proximal links are of equal length $L_{1}$ and both distal links are of equal length $L_{2}$. The base link is of null length, with both base pivots overlapping for optimal kinematics. A parallel architecture is adopted in order to reduce link inertia, thus improving safety for human interaction. Indeed, unlike traditional serial robots, actuators in parallel robots are all fixed to the base, allowing for much lighter links. The effector includes a rotating handle and wrist rest, allowing the user to interact with the robot. A trigger button, integrated into the handle, provides additional user input. The wrist rest is designed in tandem with the planar architecture to support the user’s arm against gravity by rolling along a table, ensuring more comfort during use. The planar workspace is horizontal, removing the need for gravity compensation in the design and control. The entire assembly can be securely mounted at the end of a table using clamps for stable operation.

**Figure 2:**
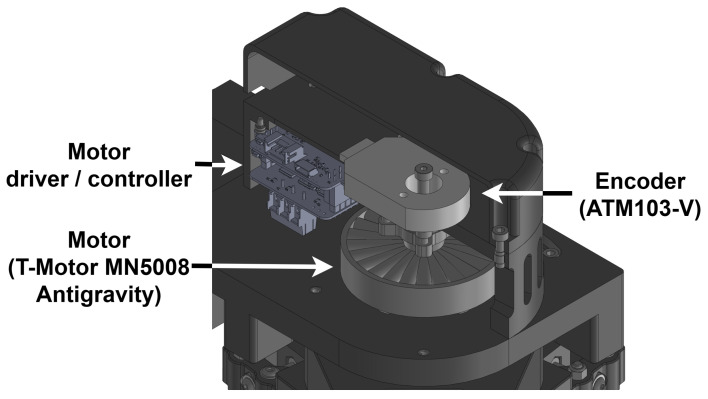
Cut view of a motor cell. An incremental encoder is installed in the back of the motor. The motor is driven by a custom-made driver/controller for BLDC motors.

As shown in Figure 2, the robot is actuated using two 14-pole-pair brushless direct current (BLDC) outrunner motors (T-Motor MN5008 Antigravity), which are off-the-shelf drone motors priced at approximately CAD 130 each. These motors feature very low rotor inertia (8500 g/mm^2^) and minimal cogging torque (≤0.009 Nm), with an approximate nominal torque of 0.42 Nm. Because of the intended application of this motor, both cogging and nominal torque values were unavailable in datasheets and were measured experimentally. Each motor is controlled by a custom driver implementing a BLDC Field-Oriented Control (FOC) algorithm. To improve torque output, a two-stage reduction system is employed, utilizing a timing belt and a Capstan drive. A 20:1 reduction ratio is used in order to keep motor transparency for backdrivability. The use of a timing belt and Capstan drive allows for smooth motion and no backlash, which are important for user interaction as well as control stability. The Capstan drive output fixture can also serve as a pivot structure for the proximal link. This design achieves high transparency output, offering performance comparable to direct-drive systems while being more compact and using smaller, more affordable actuators. With a conservative motor shutoff temperature of 80 °C, the robot can safely deliver reaction forces above 28 N across its entire workspace for up to 180 s—the same force target set for the development of the MIT-Manus [15]. In addition, the PlanAID can transiently generate forces up to 56 N.

### 2.2. Sensors

**Figure 3:**
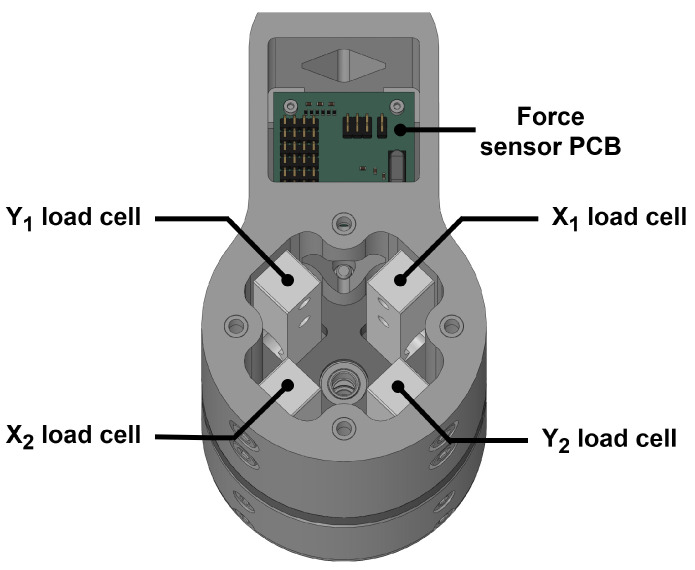
Load cell assembly of the force sensor with the PCB. Two load cells are used for each of the two horizontal axes. These two load cells are placed in opposite directions in order to cancel out interference signals from force input in the other axis.

Motion feedback is provided by CAD 30 incremental encoders (11-bit resolution ATM103-V, Same Sky, Lake Oswego, OR, USA) installed in the back of each motor, as shown in Figure 2. Effector movement can then be interpreted using forward kinematics, as shown in the kinematics sections. Force feedback is provided by a custom-made force sensor located at the end-effector directly above the handle. In addition, 10 kg load cell beams (approximately CAD 15) are used in the assembly to link the handle with the rest of the robot, thereby measuring any force input when moving the robot by using the handle. However, unlike high-cost multi-axis force sensors, these consumer-grade load cell beams are designed to measure the force in a single direction and can output a signal when subjected to bending in other directions, which introduces undesirable interference or “crosstalk”. This problem is addressed by Mayetin and Kucuk in their design of a low-cost force-sensing unit for wrist rehabilitation [16]. Following this design, two load cells are installed in opposite directions in each axis, as presented in Figure 3. This configuration ensures that the interference from non-ideal forces is cancelled out by having both load cells measure interference with opposite signs, while the desired force input is amplified, providing more accurate and reliable feedback. Signal amplification and conversion are handled by a small printed circuit board (PCB).

Because of the low-cost nature of the load cells, their output signal is quite noisy, making it difficult to use directly in a force control loop. This, however, is mitigated by applying a low-pass filter with a high cutoff frequency of 20 Hz, which reduces the noise to an adequate level while maintaining a good response time for human movement (<10 Hz).

The initial prototype bill of materials estimates the cost of the PlanAID at CAD 1200 for small-scale production, which is substantially lower than that of previously reported clinical planar robots. At this price point, the PlanAID also positions itself among low-cost alternatives such as the PLANarm2, which has an estimated cost of EUR 1521 (approximately CAD 2500) [17].

### 2.3. Kinematics

**Figure 4:**
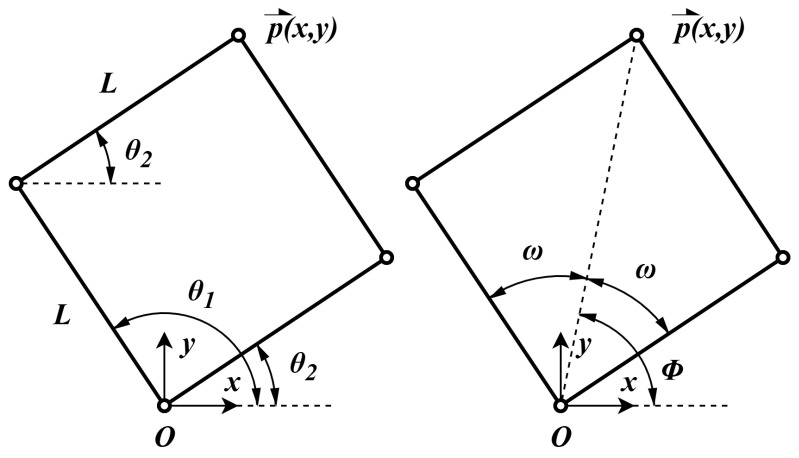
Schematic of the robot’s architecture in the desired configuration. The double pivot at *O* is fixed.

The robot architecture was chosen based on multiple factors, such as cost-effectiveness, friction, and bulkiness. In the end, a planar and symmetrical parallel robot was chosen because of its low inertia, low number of actuators, and usability with both arms. Analysis of the proximal–distal link ratio resulted in a ratio of 1 being optimal because it provides high isotropy across the desired workspace and greatly simplifies kinematics computation [9]. Therefore, all links are of length *L*. The robot architecture is presented in Figure 4.

### 2.4. Inverse Kinematics

The joint variables $\vec{\theta }={\left[\theta_{1},\theta_{2}\right]}^{T}$ can be expressed as a function of the end-effector position $\vec{p}={\left[x,y\right]}^{T}$ using geometric relations and Figure 4.

(1) $$\left[\begin{array}{c} \theta_{1}\\ \theta_{2} \end{array}\right]=\left[\begin{array}{c} \varphi +\omega \\ \varphi -\omega \end{array}\right]$$

where

(2) $$\varphi =\arctan\left(\frac{y}{x}\right)$$

(3) $$\omega =\arccos\left(\frac{\sqrt{x^{2}+y^{2}}}{2L}\right)$$

### 2.5. Forward Kinematics

The end-effector position $\vec{p}={\left[x,y\right]}^{T}$ can be expressed as a function of the joint variables $\vec{\theta }={\left[\theta_{1},\theta_{2}\right]}^{T}$ as presented in Equation (4). Since all links have the same length, it forms a parallelogram, as shown in Figure 4.

(4) $$\left[\begin{array}{c} x\\ y \end{array}\right]=L\left[\begin{array}{c} \cos\left(\theta_{1}\right)+\cos\left(\theta_{2}\right)\\ \sin\left(\theta_{1}\right)+\sin\left(\theta_{2}\right) \end{array}\right]$$

### 2.6. Jacobian Matrices

Using the closed-loop kinematic equations, the Jacobian matrix $J^{\prime }$, which relates the end-effector motion to the joint motion, can be derived as shown in Equation (5). Owing to the robot’s parallel architecture, $J^{\prime }$ is obtained from the combination of the Jacobian matrices J and K presented in Equations (6) and (7):

(5) $$J^{\prime }=J^{-1}K$$

where

(6) $$J=\left[\begin{array}{cc} x-L\cos(\theta_{1}) & y-L\sin(\theta_{1})\\ x-L\cos(\theta_{2}) & y-L\sin(\theta_{2}) \end{array}\right].$$

(7) $$\begin{array}{cc} K & =\left[\begin{array}{cc} A & 0\\ 0 & B \end{array}\right]\\ A & =L(y\cos\left(\theta_{1}\right)-x\sin\left(\theta_{1}\right))\\ B & =L(y\cos\left(\theta_{2}\right)-x\sin\left(\theta_{2}\right)) \end{array}$$

### 2.7. Workspace

All robot links are 300 mm in length. The selected user workspace is modelled as an ellipse with a major axis of 600 mm and a minor axis of 280 mm [9], yielding a total area of 0.132 $m^{2}$. In comparison, the MIT-Manus, with its SCARA architecture, provides a rectangular workspace of 15 in. × 18 in. (381 mm × 457.2 mm) [18], corresponding to a slightly larger area of 0.174 $m^{2}$. Among low-cost alternatives, the PLANarm2 features a similar elliptical workspace to the PlanAID with axis lengths of 502.75 mm and 222 mm [17], resulting in a smaller area of 0.088 $m^{2}$. In this regard, the PlanAID positions itself between low-cost and high-end options in terms of workspace size.

The kinematic analysis and workspace optimization were conducted earlier in the project and are reported in a separate article by the authors [9]. This work included a representation of the robot’s reachable workspace overlaid with the desired user workspace.

### 2.8. Control Methodology

**Figure 5:**
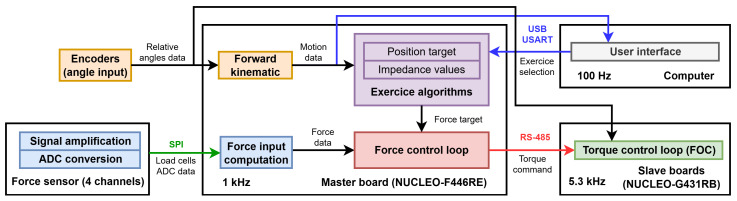
Control pipeline of the robot. Motion data are obtained from encoders, while load cells’ analog-to-digital data are obtained from the force sensor using SPI communication. The master board then computes both forward kinematics and force input to feed both the exercise and force control loops. Torque commands are sent from the master board to the slave boards through RS-485 communication. Torque control algorithms on the slave boards allow smooth torque control of the motors. The user interface allows the user to select and visualize the exercise using USB USART communication.

The control methodology is summarized with the control pipeline shown in Figure 5. This pipeline presents the different components of the robots, such as sensors and controllers, and how they interact in order to provide exercise feedback. The principal modules are the motion input (yellow), force input (blue), exercise control loop (purple), robot control loop (red), user interface (grey), and torque control loop (green).

### 2.9. Motion Input

The quadrature encoders are employed to obtain relative angle data and allow us to compute the current angles of both proximal joints (yellow, Figure 5). Using the forward kinematics of Equation (4), the current effector position can be computed, while the Jacobian matrix of Equation (5) is utilized to compute the effector velocity:

(8) $$\dot{\vec{p}}=J^{\prime }\dot{\vec{\theta }}$$

where

(9) $$\dot{\vec{\theta }}=\frac{d\vec{\theta }}{dt}$$

For Cartesian acceleration, the following approximation is used:

(10) $$\ddot{\vec{p}}\approx \frac{d\dot{\vec{p}}}{dt}$$

A low-pass filter is applied to the angular velocity and end-effector acceleration to attenuate high-frequency noise.

### 2.10. Force Input

Force input is obtained through the force sensor, which includes the PCB tasked with amplifying and converting the load cell analog signals into digital signals; these can then be transmitted to the master board via SPI communication (blue, Figure 5). For each axis, the values from both load cells are combined to cancel out interference, as previously stated. The resulting signals are then converted to Newtons using the conversion matrix S, as presented in Equation (11). Since the force sensor is fixed to the distal link, data are rotated from sensor referential to robot referential using rotation matrix $R_{r}^{s}$ to obtain the force input ${\vec{f}}_{s}$:

(11) $${\vec{f}}_{s}=R_{r}^{s}S\left[\begin{array}{c} f_{x1}+f_{x2}\\ f_{y1}+f_{y2} \end{array}\right]$$

(12) $$R_{r}^{s}=\left[\begin{array}{cc} \cos(\theta_{2}-\frac{\pi }{4}) & -\sin(\theta_{2}-\frac{\pi }{4})\\ \sin(\theta_{2}-\frac{\pi }{4}) & \cos(\theta_{2}-\frac{\pi }{4}) \end{array}\right]$$

where $f_{x1}$, $f_{x2}$, $f_{y1}$, and $f_{y2}$ are the load cell #1 and load cell #2 digital signals for axes *x* and *y* in the force sensor referential.

### 2.11. Impedance Control

The PlanAID robot uses an impedance control scheme, which is used in several compliant human–robot interactions in industrial robots [19]. As the name suggests, impedance control aims to control the robot’s mechanical impedance to simulate a virtual object in a mass, spring, and damper system [20]. This system can be expressed by the following equation for 1 DoF, where *m* is the mass, *c* is the damping constant, and *k* is the spring constant:

(13) $$f=m\ddot{x}+c\dot{x}+kx$$

Mechanical impedance is controlled by computing the desired force output using motion input and applying it at the robot effector. Furthermore, compliant robot motion is achieved by modifying the desired position $x_{d}$ and velocity ${\dot{x}}_{d}$ in the following equation:

(14) $$f_{d}=-m\ddot{x}-c\left(\dot{x}-{\dot{x}}_{d}\right)-k\left(x-x_{d}\right)$$

### 2.12. Exercise Algorithms

As previously mentioned, exercises are programmed using impedance control. The selection of exercises is handled through the user interface, which transmits commands via USB USART. Based on motion data, the algorithm (purple in Figure 5) uses position target and impedance gains, including fixed spring and damper constants, to compute the force target for the force control loop.

**Figure 6:**
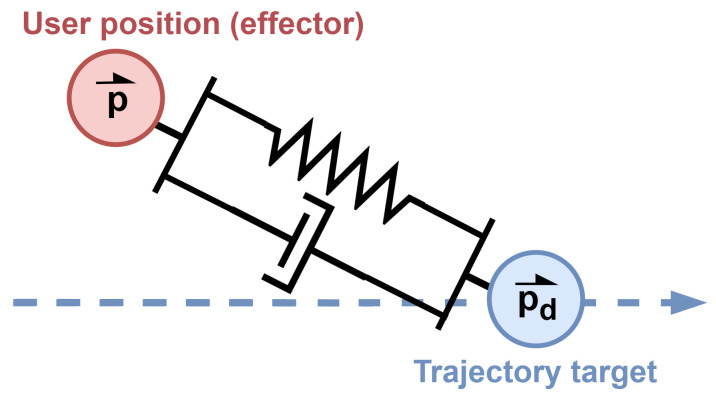
Representation of passive exercise control. A virtual spring and damper connect the user position to the target position along the trajectory (dotted blue arrow) in order to move the effector in the desired direction with the desired level of assistance.

Passive exercise control is achieved using guided trajectories, during which the robot assists the user’s movement by outputting a force in the desired direction of movement [13]. This approach is particularly useful in maintaining mobility or supporting users in the early stages of recovery. Impedance control is used with a high virtual spring value in order to follow a target along the trajectory, as shown in Figure 6.

To prevent instability and reduce overshoot associated with high stiffness, virtual damping is also incorporated. Both stiffness and damping parameters are adjustable, allowing the customization of assistance levels based on individual user needs. The target position progresses along the predefined trajectory at a configurable speed. If the user resists movement and the end-effector deviates significantly from the target, the target position halts until the resistance is released. This ensures safe and responsive interaction. The desired force output ${\vec{f}}_{d}$ is then computed as in Equation (15) following Equation (14) in two dimensions. No virtual mass is included in passive exercises, as it would only introduce response delays and potential overshoot. Therefore, it is simply set to zero.

(15) $${\vec{f}}_{d}=-K\left(\vec{p}-{\vec{p}}_{d}\right)-C\dot{\vec{p}}$$

where K and C are the spring constant and damping constant matrix, respectively, described in Equation (16). These matrices are diagonal and simply contain the values for the *x*- and *y*-axes, respectively.

(16) $$K=\left[\begin{array}{cc} k_{x} & 0\\ 0 & k_{y} \end{array}\right],C=\left[\begin{array}{cc} c_{x} & 0\\ 0 & c_{y} \end{array}\right]$$

**Figure 7:**
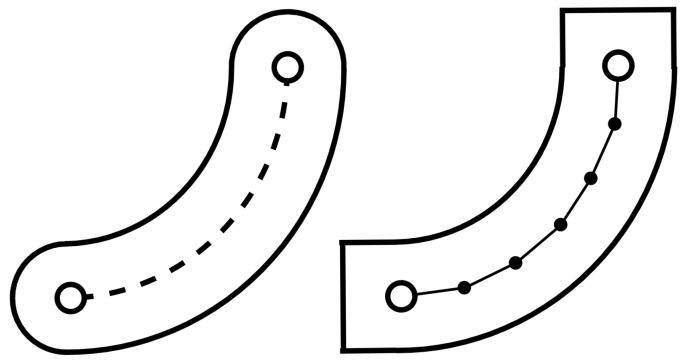
Representation of continuous virtual wall guidance (**left**) and actual virtual wall guidance (**right**). The desired trajectory, shown with a dash line, is enclosed by a virtual wall. The actual virtual walls are implemented by discretizing the trajectory into smaller, linear wall segments (dotted line).

Active exercise control is achieved using virtual wall guidance in a strategy named tunnelling [21]. In this method, the user is free to move the effector within a defined channel, surrounded by virtual walls, which guide the movement by preventing the user from diverging from the intended path. Figure 7 illustrates this concept by presenting continuous virtual wall guidance on the left, as well as the method used to program the path on the right. The intended trajectory is discretized into smaller linear segments, each bounded by virtual walls. By using sufficiently high discretization, the virtual walls remain tangent to the trajectory at all points, ensuring continuous guidance.

**Figure 8:**
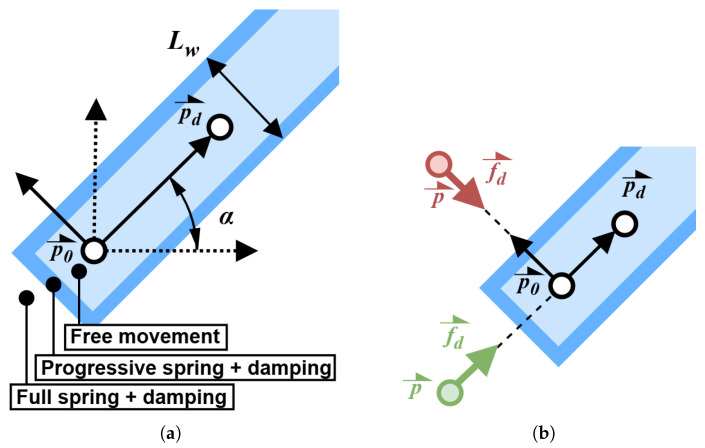
(**a**) Referential used to program virtual walls between points ${\vec{p}}_{0}$ and ${\vec{p}}_{d}$ of a discretized virtual wall path. The dotted axis represents the robot’s referential, while the full-line axis represents the virtual wall’s referential. Virtual walls are implemented with zones of progressively increasing and complete gain. (**b**) Desired force ${\vec{f}}_{d}$ is applied towards ${\vec{p}}_{0}$ when moving backwards (green) or perpendicular to the desired direction (red).

Figure 8 represents the methods used to program the virtual walls between two points. The dotted and full-line axes represent the robot and virtual wall reference frames, respectively. The user’s goal is to move in the direction of ${\vec{p}}_{d}$. If the user moves too far away in any other direction, whether orthogonal to the desired path or backward, a virtual wall is reached. Virtual walls are simulated by adding high virtual spring and damping values to the command, which recreates the sensation of hitting a physical barrier. It is important to note that the damping is only applied if the user moves away from the indented trajectory, meaning that no damping is present while returning to the centre of the path. Similarly, the virtual spring pushes the user’s hand in the direction of the desired trajectory, as in Figure 6. The handle position and velocity are computed in the virtual wall referential using the following rotation matrix in Equation (17).

(17) $$R_{w}^{r}=\left[\begin{array}{cc} \cos(\alpha ) & \sin(\alpha )\\ -\sin(\alpha ) & \cos(\alpha ) \end{array}\right]$$

In order to ensure smooth interaction with the virtual walls, a small transition zone precedes the full wall in which the spring and damping values increase as the user moves progressively closer to the wall boundary. This gradual buildup prevents large jumps in spring and damping values, which could otherwise cause instability. The user interface provides the therapist with the ability to adjust key parameters, including the virtual spring and damping values, as well as the path width $L_{w}$. Virtual wall force feedback ${\vec{f}}_{d}$ is computed using Equation (18).

(18) $${\vec{f}}_{d}={R_{w}^{r}}^{-1}\left(-KR_{w}^{r}\left(\vec{p}-{\vec{p}}_{d}\right)-CR_{w}^{r}\dot{\vec{p}}\right)-M\ddot{\vec{p}}$$

where the values of matrices K and C are adjusted in both wall referential axes according to the user position, as presented in Figure 8. As for the mass, it is constant in the robot referential, as presented in Equation (19).

(19) $$M=\left[\begin{array}{cc} m_{x} & 0\\ 0 & m_{y} \end{array}\right]$$

The resulting approach creates a tunnel-like region within which the user is free to move up to the wall boundaries and is encouraged to progress in the desired direction. This strategy is similar to that proposed in the recent literature [22], where corrective forces are applied to the effector to guide it toward the centre of the trajectory. In the present work, however, a deliberate allowance is introduced before applying corrective forces, giving the user the opportunity to self-correct prior to assistance. The channel width $L_{w}$ is adjustable, with a width of zero reproducing the strategy reported in [22].

### 2.13. Force Control Loop

**Figure 9:**
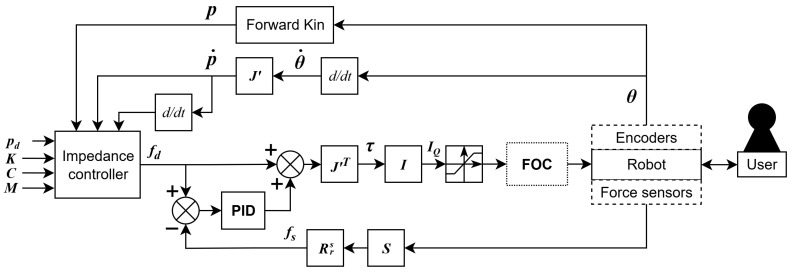
Control diagram of the robot.

Torque commands are generated in a force control loop (red in Figure 5) using force data, along with the force target, which is better presented using a control diagram (Figure 9). As previously explained using the control pipeline (Figure 5), encoder angles $\vec{\theta }$ are used to compute the Cartesian position $\vec{p}$, velocity $\dot{\vec{p}}$, and acceleration $\ddot{\vec{p}}$ for the controller using forward kinematics (Equation (4)) and a Jacobian matrix $J^{\prime }$ (Equation (5)). The desired force ${\vec{f}}_{d}$ is computed using the impedance controller according to motion input and exercise inputs ${\vec{p}}_{d}$, K, C, and M. Force input ${\vec{f}}_{s}$ is also computed using conversion matrix S and rotation matrix $R_{r}^{s}$ (Equation (12)).

Since the robot has low inertia and is not affected by gravity, compensation for robot inertia, gravity, and Coriolis and centrifugal forces is not included in the command loop. Indeed, these compensation matrices, such as the inertia matrix, are complex to compute on embedded systems and offer little benefit in this case. Furthermore, actuator friction acts as a damper for inertia effects.

To ensure smooth motion and compensate for friction, a force-based PID controller is implemented, comparing the desired force with the measured input force (Figure 9). This approach enables the controller to accurately apply the commanded force at the end-effector by correcting any offsets. In the case of free movement, the desired force is set to 0, such that any measured input force is fed into the PID controller to compensate for friction. The resulting output force is then mapped into the desired joint torques $\vec{\tau }$ using the Jacobian matrix:

(20) $$\vec{\tau }=J^{\prime T}{\vec{f}}_{d}$$

### 2.14. Torque Control Loop

As presented in Figure 9, desired torques are converted into the current target ${\vec{I}}_{q}$ using conversion matrix I, which was experimentally determined using a high-precision force sensor (ATI mini45). These current targets are then processed through a saturator to ensure that they remain within the operational limits of the system before being sent to the motor drivers (green, Figure 5). These command loops operate at a frequency of 1 kHz on the master control board.

Unlike brushed motors, electric commutation in BLDC motors must be executed through an algorithm that controls the voltage across the motor’s three phases to rotate the magnetic field. Many such algorithms exist, ranging in complexity. However, simpler ones like trapezoidal control are a better fit for sensorless speed control, such as RC drone applications. A Field-Oriented Control (FOC) algorithm, on the other hand, allows for smoother, less noisy operation and aims to maximize the global torque output, making it better fitted for torque control [23]. However, this performance is counterbalanced by high complexity and high-frequency computation. For this reason, the algorithm is run on separate slave boards at high frequency. This algorithm was adopted using the STM32 MotorControl Workbench (MCSDK6.4.1, STMicroelectronics, Geneva, Switzerland) [24].

### 2.15. User Interface

**Figure 10:**
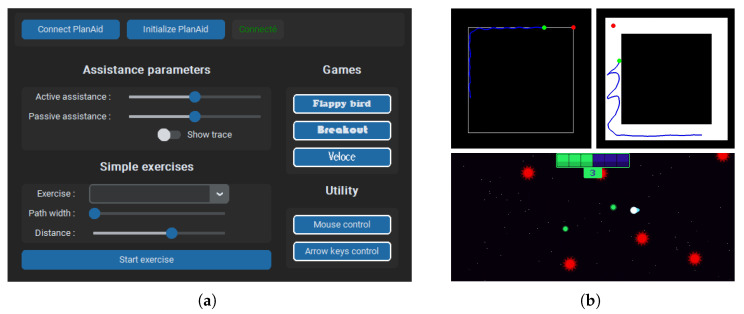
(**a**) The robot user interface. The top buttons allow one to connect and initialize the robot. The left column allows the user to select simple exercises, as well as adjusting parameters such as the path width, showing the user’s movement trace, and changing the assistance level. The right column contains games and utility programs to allow the user to control mouse or keyboard input using the robot. (**b**) Visual representation of passive exercise (upper left), active exercise (upper right), and game exercise (bottom) in the GUI. The blue line and red dot in the upper figures represent the user trajectory and next objective.

A user interface (grey, Figure 5), presented in Figure 10a), was programmed in Python 3.0 using the Tkinter library to allow easy control over the robot and act as a visual interface for repetitive movement exercises. The types and parameters of exercises can be chosen at the bottom left. Exercise assistance settings such as spring and damping can be fine-tuned according to the user’s specific needs. Additional options, such as displaying the user’s movement trace, can be toggled for real-time feedback, as shown in Figure 10b). To encourage consistent participation, simple games are incorporated into the interface, providing a more playful and engaging way for users to perform exercises more frequently. Additionally, mouse and keyboard arrow control functionalities have been integrated, enabling the robot to interact with the computer’s inputs. This feature allows users to play simple computer games using the robot, offering a novel form of rehabilitation without the need to pre-program specific games into the user interface.

### 2.16. Force Sensor Validation

The robot’s functions are evaluated through experimental validation using an external high-precision force–torque sensor (ATI mini45, ATI Industrial Automation, Apex, NC, USA). The specific functions assessed include the PlanAID sensor input, the robot’s backdrivability, friction compensation, the impedance controller, and the tunnelling algorithm. Additionally, to assess less tangible characteristics such as movement fluidity, an additional qualitative evaluation of the robot’s performance is conducted via a questionnaire.

**Figure 11:**
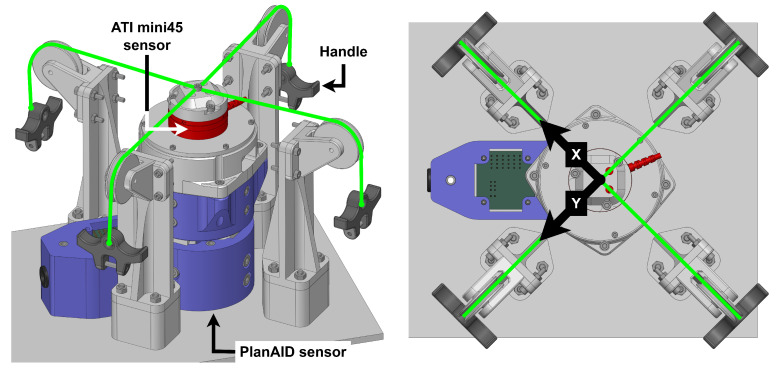
Force sensor validation setup. The PlanAID sensor (blue) is mounted on a rigid base together with a four-pulley rope-guiding structure. An ATI mini45 sensor (red) is mounted in series with the PlanAID sensor to measure the forces applied to the PlanAID sensor. The measurement axes are shown on the right. Ropes (green), routed through the pulleys, enable the application of forces with well-defined magnitudes and directions.

The friction compensation and force correction implemented in the force control loop rely on accurate force input to operate effectively. To evaluate the force sensor, an experimental setup was developed to enable the application of forces in specific directions while allowing the measurement of sensor crosstalk. This setup is shown in Figure 11. The mini45 sensor is mounted at the end of the PlanAID sensor, enabling the simultaneous measurement of the applied forces by both sensors. Ropes attached to the mini45 sensor and routed through pulleys allow forces to be applied in well-defined directions. Forces can be applied by hand using the handles or by attaching weights.

Three evaluations are performed, each with a distinct objective:

- Evaluation of the sensor output under dynamic, user-applied forces;
- Evaluation of measurement error and crosstalk under static loading;
- Evaluation of signal quality through noise density measurements.

The first test aims to characterize the sensor output under slow, low-frequency oscillatory inputs representative of human movement. Forces are manually applied along a single axis using the handles at different excitation frequencies. The objective is to evaluate crosstalk on the non-excited axes, as well as the amplitude response and phase delay of the sensor by comparing its output to reference measurements provided by the mini45 sensor.

The second test aims to quantify measurement error and crosstalk using static, discrete force inputs applied along both axes and in both directions. Forces are applied in increments of 10 N over a range of ±50 N. Measurements are recorded from the PlanAID sensor, with the mini45 sensor serving as a reference for the applied force. For each force level, five measurements are collected and averaged. Data from both the excited and unexcited axes are analyzed to evaluate the measurement accuracy and crosstalk.

Force sensor noise is further evaluated through noise density analysis. The noise density is computed using Welch’s power spectral density (PSD) estimate applied to a no-load measurement sampled at 1 kHz. This approach yields a spectral density estimate over the frequency range from 0 Hz to $f_{s}/2$, where $f_{s}$ is the sampling frequency. The noise density spectrum is then obtained by taking the square root of the PSD. This method is applied to three different signals, the mini45 signal, the sensor raw signal, and the filtered sensor signal, in order to compare the raw signal quality with that of a high-precision sensor and to evaluate the low-pass filter efficacy.

### 2.17. Backdrivability and Force Compensation Evaluation Methodology

To increase the force output, a 20:1 reduction was incorporated into the design. This choice, however, also introduced higher friction and inertia, which negatively affect the desired backdrivability. Backdrivability and force compensation are therefore evaluated using a least-squares identification method to estimate the end-effector inertia and friction coefficients. All parameters are identified from user-driven movements performed along both axes and around the centre of the workspace. Motion along a single axis is described by the following model:

(21) $$y\left(k\right)={\vec{\phi }}^{T}\vec{\theta }+e\left(k\right)$$

where *y* represents the user input force *f*, $\vec{\phi }$ represents the movement data, and $\vec{\theta }$ represents the parameters to identify, as presented in Equation (22). *e* represents the error to minimize.

(22) $$\begin{array}{c} y(k)=f(k);\\ \vec{\phi }={\left[\begin{array}{c} \ddot{x}\left(k\right),\dot{x}\left(k\right),sign\left(\ddot{x}\left(k\right)\right) \end{array}\right]}^{T}\\ \vec{\theta }={\left[\begin{array}{c} m,f_{v},f_{c} \end{array}\right]}^{T} \end{array}$$

where *m* is the mass inertia, $f_{v}$ is the viscous friction constant, and $f_{c}$ is the Coulomb friction constant. Using a dataset of *n* observations collected from back-and-forth movements at varying frequencies, the least-squares method was applied to identify the backdrivability values according to Equation (23):

(23) $$\vec{\hat{\theta }}={\left(\Phi^{T}\Phi \right)}^{-1}\Phi^{T}\vec{Y}$$

where

(24) $$\begin{array}{c} \Phi^{T}=\left[\begin{array}{c} {\vec{\phi }}^{T}\left(1\right)\\ \vdots \\ {\vec{\phi }}^{T}\left(n\right) \end{array}\right]\\ \vec{Y}={\left[\begin{array}{c} y(1),\ldots ,y(n) \end{array}\right]}^{T} \end{array}$$

Parameters are obtained for both axes from uncompensated movements, meaning that the motors were turned off, allowing the measurement of the mechanical backdrivability performance. The same evaluation is then repeated with force compensation enabled to assess its effectiveness in improving the backdrivability. For force compensation, only a $k_{p}$ gain of 1.6 is used. All parameter identifications are carried out using dedicated identification data and subsequently validated on a separate dataset.

### 2.18. Impedance Controller Validation and Stability Analysis Methodology

The impedance controller is evaluated in terms of both functionality and stability using the robot itself. Controller functionality is demonstrated through two exercises, a constant-target task and an active square-tunnel task, which, respectively, represent passive and active exercise modes. The objectives are to verify that the impedance controller computes the desired force correctly and to compare the target force with the user-applied force in order to confirm that the intended interaction force is perceived by the user. For the constant-target task, the target is positioned near the centre of the workspace with a spring constant of 0.2 N/mm and a damping constant of 0.02 Ns/mm. Horizontal and vertical oscillatory movements are performed to assess the controller response along both axes. For the square-tunnel task, multiple revolutions are executed with three objectives: maintaining the path centre, following the outer boundary, and following the inner boundary. These conditions allow the evaluation of both free motion and wall interaction feedback provided by the controller. In this exercise, a wall spring constant of 10.5 N/mm and a wall damping constant of 0.085 Ns/mm are used.

As previously mentioned, the impedance parameters can be adjusted through the user interface using sliders for both passive and active training modes. These sliders allow the modification of the spring and damping constants, as defined in Equation (16). The controller’s stability is then evaluated by identifying the maximum stable spring constant for different damping values. This preliminary evaluation is intended to provide an overall assessment of the system’s stability margins in the context of its development.

Stability is assessed through user-induced perturbations performed by a healthy subject, with a constant target located at the centre of the workspace and force compensation enabled using a $k_{p}$ gain of 1.6.

### 2.19. Tunnelling Controller Validation Methodology

The tunnelling control used in active exercises is validated by comparing distance and force data normal to the desired direction of movement in a linearly guided trajectory, similarly to what is presented in Figure 8. This comparison is intended to showcase the tunnelling controller’s reaction to the user nearing the virtual wall and the user’s interaction with the wall.

### 2.20. Users’ Perceptions Evaluation Methodology

Following the experimental validation of the robot’s functions, a subjective evaluation of the device is conducted with healthy participants. This evaluation is not intended to validate the robot’s performance in a real rehabilitation setting but rather to gather feedback from individuals other than the designers to guide further development. Participants are asked to report their levels of agreement with 6 statements, such as ease of use and control smoothness, using a 5-point Likert scale, where 1 means “entirely disagree”, 5 means “entirely agree”, and 3 is a neutral option (neither agree nor disagree). User comments are also collected for a clearer qualitative evaluation. The questionnaire is completed after a pilot test of the fully functional prototype using basic exercises and games, as presented in Figure 10b. All 9 participants (ages 24–39, 6 men, 3 women) are members of Laval University’s robotic laboratory, with none of them having any upper-limb sensorimotor impairments.

Each participant is first given a brief introduction to the robot, including its purpose, functionality, and main features, delivered by an assistant. Participants are then allotted several minutes to familiarize themselves with the device. Subsequently, the assistant instructs the participant to operate the robot by initiating it and launching various types of exercises. During these exercises, participants are asked to observe and mentally take note of different interaction aspects, such as movement smoothness and assistance fluidity. Upon completion of the tasks, the participant is asked to complete a form and may include additional notes if desired.

This qualitative evaluation is intended to capture participants’ subjective perceptions of robot functionalities that are difficult to quantify, such as interaction smoothness and ease of use. The results are not intended to reflect the device’s effectiveness in real-world rehabilitation settings.

## 3. Results

### 3.1. Force Sensor

**Figure 12:**
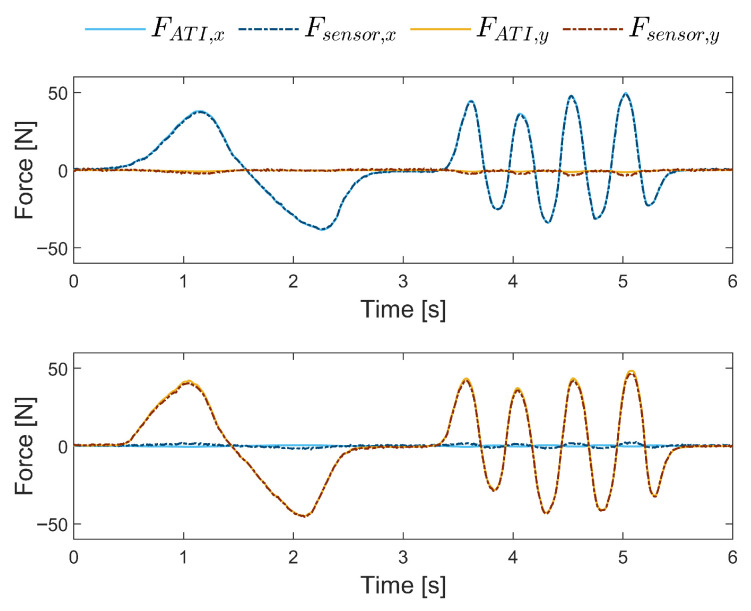
Comparison of force input from the PlanAID low-cost sensor and the mini45 high-precision sensor for force applied in the *x*- and *y*-axes during slow and low-frequency oscillating application.

Figure 12 shows the application of a force along the *x*- and *y*-axes separately under slow low-frequency oscillatory inputs. At these low frequencies, representative of human movement, the PlanAID sensor is able to closely reproduce the mini45 sensor output with minimal error and crosstalk.

The mean static force measurements for both the *x*- and *y*-axes are presented in Appendix A. The measurement error is computed by comparing the sensor output with the mini45 reference measurements, while crosstalk is evaluated by taking the absolute error measured on the unexcited axis and dividing it by the mini45 reading on the excited axis. Table 1 presents the compiled results of these error computations. From these results, it can be observed that the sensor error remains minimal, not exceeding 5%, which demonstrates sufficient precision for this application. Crosstalk is somewhat higher, particularly in the −x and +y directions compared to the others. Nevertheless, the approximate mean crosstalk of about 3% is very low and does not significantly impact force compensation. These results highlight that the use of multiple load cells is effective in substantially reducing crosstalk.

**Figure 13:**
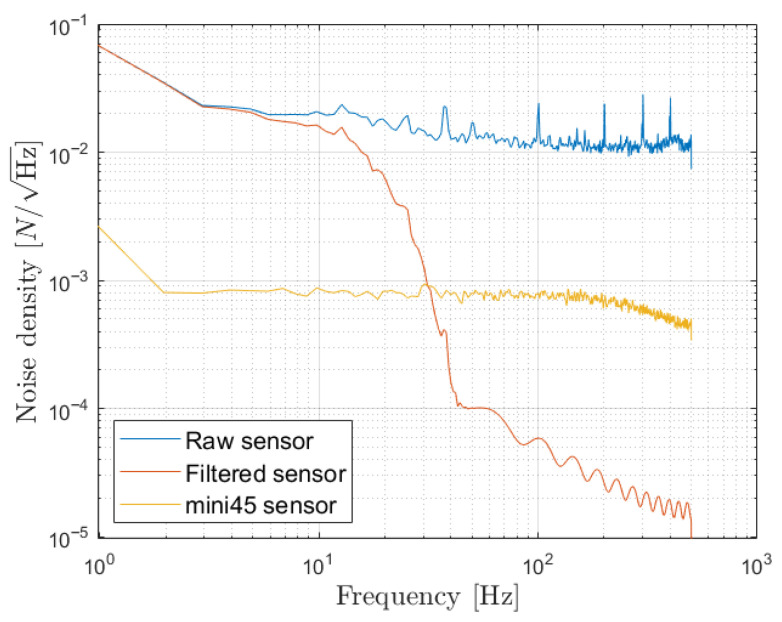
Noise density spectrum for raw sensor, filtered sensor, and mini45 sensor signals using a no-load measurement at 1 kHz.

Figure 13 presents the noise density spectrum for both the raw and filtered force sensor signals, as well as for the mini45 high precision sensor. From these results, we can observe that the raw sensor signal exhibits noticeable noise, with a mean noise density of $0.0126\;N/\sqrt{\mathrm{Hz}}$, which is expected for a low-cost alternative. For comparison, the mini45 sensor yielded a noise density on the order of $10^{-3}\;N/\sqrt{\mathrm{Hz}}$. However, by applying the 20 Hz cutoff low-pass filter, noise above 20 Hz is greatly attenuated, resulting in a mean noise density comparable to that of the mini45 sensor.

### 3.2. Backdrivability and Force Compensation

Using the identification data, the parameters presented in Table 2 were obtained and subsequently validated using the validation datasets. All identification and validation data are presented in Appendix B. Without active force compensation generated from the force PID (Figure 9), the reflected inertia is approximately 1.1 kg, with average friction constants of 4.45 N/(m/s) and 1.91 N. For comparison, the MIT-Manus reports “Coulomb friction less than 2 N, viscous damping less than 4 N/(m/s), and inertia less than 1.7 kg” [25]. This indicates that the PlanAID achieves backdrivability comparable to that of a direct-drive robot, despite the inclusion of a 20:1 reduction ratio. This can be attributed to the choice of motors with low rotor inertia and minimal cogging torque.

With force compensation enabled, all parameters show a notable decrease. The effective mass inertia is reduced by around ∼27.3%, while viscous damping is reduced by ∼70%. Most importantly, the reflected static friction is reduced by nearly ∼91.1%, thereby enabling the smoother execution of small movements.

### 3.3. Impedance Controller Validation and Stability Analysis

**Figure 14:**
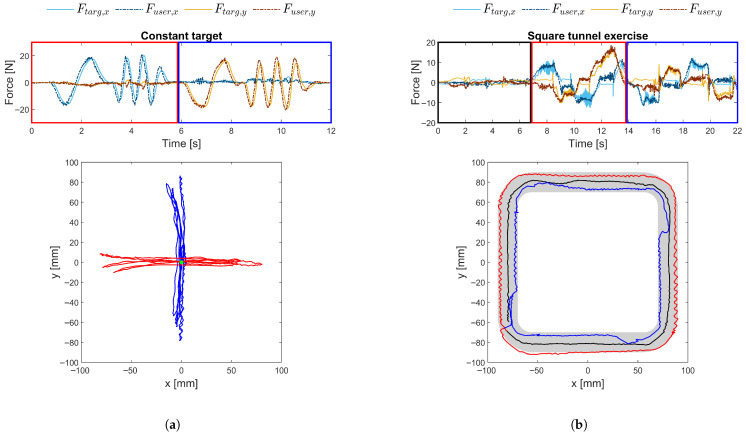
(**a**) Comparison between the impedance controller’s target force and the resulting user-applied force for a constant target at the centre of the workspace (green dot), with a spring constant of 0.2 N/mm and a damping constant of 0.02 Ns/mm. Horizontal (red) and vertical (blue) movements are shown, with the user position displayed in the lower graph. (**b**) Comparison between the impedance controller’s target force and the resulting user-applied force during an active square-tunnel exercise, with a spring constant of 10.5 N/mm and a damping constant of 0.085 Ns/mm. The three objectives are maintaining the path (black), following the outer boundary (red), and following the inner boundary (blue), as shown in the lower graph. The path boundaries are indicated in grey.

Figure 14 presents a comparison between the user’s input force and the target force generated by the controller in the two scenarios. In the constant-target scenario, the output force aligns closely with the desired force along both axes, confirming effective controller performance. A slight response delay is observed as the robot reacts to user deviations from the target position, requiring the user to overcome residual uncompensated friction and inertia.

For the active square-tunnel exercise, the output force similarly follows the target force, particularly near the tunnel walls, where force feedback is applied. In regions where the target force is zero, corresponding to free movement within the tunnel, some user-applied input force is still detected due to the same remaining uncompensated friction and inertia. This residual force remains small, typically below 3 N.

**Figure 15:**
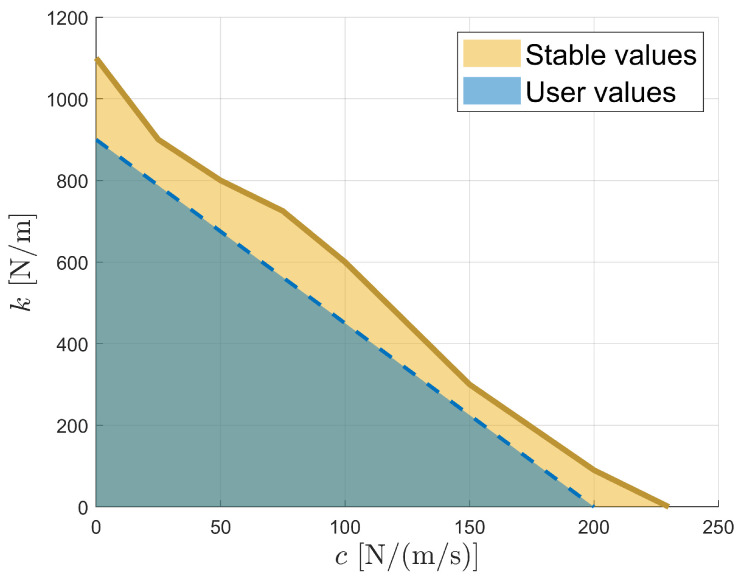
Representation of the zone of stable constant values and the range of user values accessible in the user interface.

The stability analysis results are presented in Figure 15. When applied without the other, the spring constant can reach a value of 1100 N/m and the damping constant can reach 230 Ns/m before instability occurs. Typically, spring constants are limited when combined with low damping values, resulting in reduced *k* values for *c* near zero. However, this does not appear to be the case for the PlanAID, likely due to the inherent damping provided by mechanical friction. When the spring and damping are combined, the overall stability is reduced, requiring both parameters to be decreased to maintain safe operation. By comparison, the MIT-Manus controller allows stiffness of 2000 N/m without damping [18], making it significantly more stable than the PlanAID.

This loss of stability is an expected consequence of cost reduction: lower-precision sensors and economical production methods contribute to lower overall stability. Notably, the use of 3D-printed parts for the robot base and links reduces the mechanical stiffness, introducing unwanted elasticity into the mechanism. Similarly, a key contributor to system stability is the controller. In this case, the controller is integrated directly with the robot, necessitating the use of a microcontroller rather than a full computer. The effects of cost reduction are also reflected in the wall guidance behaviour, as illustrated in Figure 14b. When moving along a virtual wall while applying a force against it, the interaction results in oscillatory motion in which the user repeatedly bounces off the wall, as evidenced by the red and blue trajectories. This behaviour is detrimental to user interaction and indicates reduced stability margins.

Despite these limitations, in practice, the simulated stiffness generally remains below 400 N/m [25], which is sufficient to simulate virtual walls—a capability that is achievable, as shown by the results. To ensure safe operation, assistance parameters are restricted to the blue zone in Figure 15, providing a margin of safety before instability occurs.

### 3.4. Tunnelling Validation

**Figure 16:**
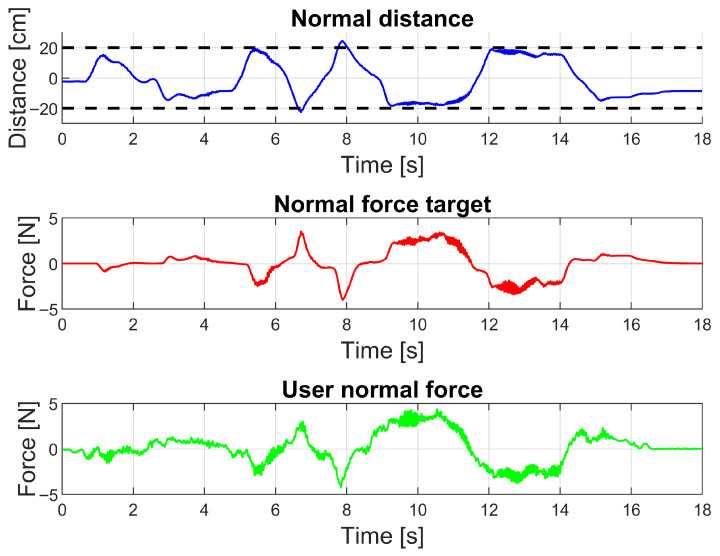
Normal distance and force data for a walled trajectory in active exercise mode. The top graph presents the normal distance from the centre of the desired path. The dotted lines represent the distance to the virtual walls. The middle and bottom graphs present the target and user normal forces. Spring and damper force is applied when nearing the wall and increases with distance from the wall. User force mirrors target force.

Tunnel interaction is presented in Figure 16. In it, the controller applies force when the user approaches the virtual walls, and this force increases rapidly when the user moves beyond the tunnel boundaries. This behaviour is reflected in the resulting user force, which closely mirrors the force target as desired.

### 3.5. Users’ Perceptions

**Figure 17:**
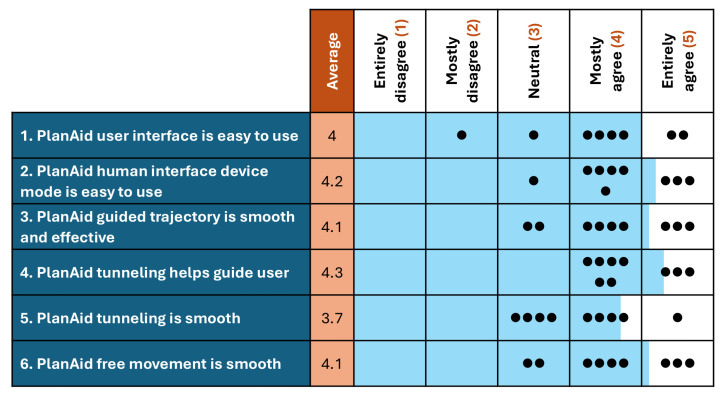
Results of the questionnaire completed by each participant after utilizing the functions of the robot. The dots represent the scores given by each participant.

The qualitative questionnaire results are presented in Figure 17. Participants found the user interface easy to use (Q1, AVG: 4) but recommended small adjustments, such as streamlining the initialization process. The human interface device mode (mouse and keyboard control) was well perceived (Q2, AVG: 4.2), with the only complaint being that the input deadzone was too large and felt unusual to use. Participants found that the guided trajectory was effective (Q3, AVG: 4.1) but that the movement felt somewhat “grainy”, as if moving through sand. For the active tunnelling control, participants found the virtual walls effective in guiding the user (Q4, AVG: 4.35) but that sharp transitions in the direction of the tunnel often led to large spikes in force output (Q5, AVG: 3.7). For active free movement, friction compensation proved effective at medium to high speeds but appeared somewhat “jumpy” at low speeds (Q6, AVG: 4.1), likely due to the robot repeatedly overcoming static friction in close succession. These results indicate that the users’ perceptions in the selected group were good. However, some aspects, such as control smoothness, should be improved.

### 3.6. Limitations

The backdrivability and force compensation evaluations were performed around the centre of the workspace, where the user is expected to carry out most tasks. Since the robot’s dexterity is not uniform across the entire reachable workspace, the Cartesian inertia and friction vary with the position. However, because dexterity remains high throughout the user workspace [9], the obtained parameters can be considered representative mean values and can be indicative of overall performance for comparison to other devices.

The stability analysis was conducted using perturbations applied by a single healthy participant. While the estimated stable state was conservative, this approach does not account for factors commonly encountered in rehabilitation, such as spasticity and variations in limb impedance, which can significantly influence stability. Indeed, user participation and range of motion can drastically alter limb impedance, resulting in substantial variations in resistance [26]. Likewise, tremor or spasticity introduces perturbations into the system [27], and such unpredictable movements—particularly involuntary movements—can generate spikes in the impedance controller output force. Further testing is therefore required, including evaluations of robot stability when interacting with environments of varying stiffness [18]. Additionally, adapting the impedance control strategy to accommodate variable stiffness and damping should be considered [26,27]. Additionally, long-duration interactions should also be tested.

This work presents the development of a new rehabilitation robot technology. However, the validation data presented are not indicative of its effectiveness in helping rehabilitation patients. Indeed, no clinical trials were performed and the user data obtained from healthy participants only aimed to offer qualitative feedback on the robot interaction to guide the development process. The experiments aimed to validate the functionality of the robot and its features, as well as comparing its performance with that of other rehabilitation robots.

Future work will focus on further developing the prototype with the aim of conducting formal clinical trials. Usability and training effectiveness will be specifically addressed by integrating feedback obtained from these trials with input from healthcare professionals. Additional improvements, such as increased stability margins, considering the discussed challenges, are also required before the robot can be considered suitable for real-world rehabilitation applications.

## 4. Conclusions

Due to the rising incidence of disability and the decreasing availability of healthcare professionals, the development of robot-assisted rehabilitation has seen significant progress. Such technology holds the potential to deliver easy access to early-stage, repetitive, and intensive rehabilitation exercises that are critical for optimal recovery. However, current rehabilitation robots are often limited to clinical use due to their high cost and bulky design. This paper presents the development of a control strategy for the PlanAID robotic prototype, which enables both passive and active exercise modes, implemented through a force control scheme using an impedance controller.

The prototype offers at least 28 N of force across the workspace with high motor transparency through the use of low-cost outrunner BLDC motor and a two-stage 20:1 Capstan and timing belt reduction. Force feedback is provided by a low-cost multi-axis force sensor using load cell beams and allows for friction compensation using a force PID. Motor driving is implemented using an FOC control loop on separate driver microcontrollers to operate at very high frequencies and offer good torque performance with smooth motion. Both passive and active exercises are programmed using virtual springs and dampers, effectively simulating pulling forces and virtual walls.

Experimental validation demonstrates that the PlanAID robot could potentially provide both passive and active rehabilitation exercises using cost-effective components while maintaining reliable force output. Future work will focus on translating this technology to real-world rehabilitation settings and evaluating its effectiveness in clinical trials. This process will begin with further testing of stability and safety margins, followed by the development of a more user-friendly version of the robot and improvements to the user interface through the integration of targeted exercises. These exercises will be designed in collaboration with rehabilitation professionals and validated with participation from individuals living with upper-body disabilities.

## Figures and Tables

**Table 1:** Mean and maximum sensor and crosstalk errors obtained from a set of discrete force measurements in the range of ±50 N for both *x*- and *y*-axes. Maximum error is shown in parentheses.

Axis	Sensor Error	Crosstalk Error
*x*	1.84% (3.04%)	3.54% (6.60%)
*y*	2.85% (4.66%)	2.35% (5.81%)

**Table 2:** Identification results of backdrivability parameters for uncompensated (motor turned off) and force-compensated movement on both axes. The parameters are mass *m*, viscous damping $f_{v}$, and Coulomb friction $f_{c}$.

	*m* [kg]	$f_{v}$ [N/(m/s)]	$f_{c}$ [N]
Uncompensated *x*-axis	1.09	5.21	1.60
Uncompensated *y*-axis	1.11	3.70	2.22
Compensated *x*-axis	0.78	1.59	0.13
Compensated *y*-axis	0.82	1.08	0.21

**Figure A1:**
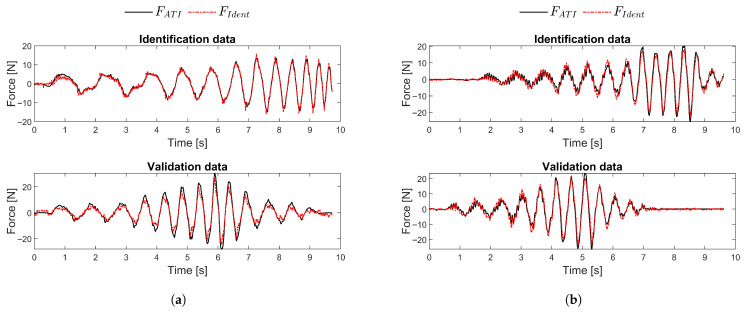

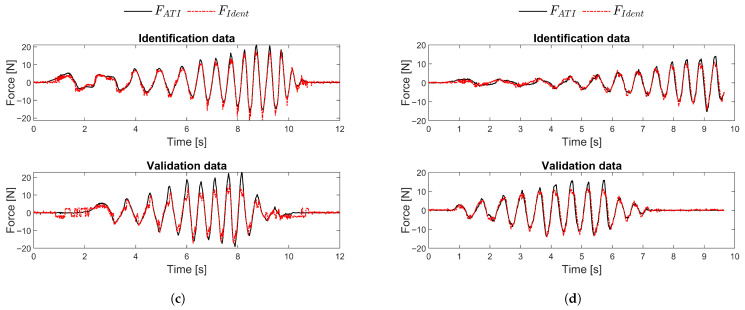
Comparison of the inertia and friction model with identification and validation data for both axes with and without force compensation enabled. The graphs show oscillatory movement for (**a**) uncompensated *x*-axis, (**b**) compensated *x*-axis, (**c**) uncompensated *y*-axis, and (**d**) compensated *y*-axis.

## Data Availability

The original contributions presented in this study are included in the article. Further inquiries can be directed to the corresponding author.

## Abbreviations

The following abbreviations are used in this manuscript:

DoF	Degrees of freedom
BLDC	Brushless direct current
CAD	Computer-aided design
FOC	Field-Oriented Control
PCB	Printed circuit board

## Appendix A

**Table A1:** Mean force input measurements on the force sensor and the ATI mini45 for forces in the range of ±50 N along the *x*-axis.

$F_{\mathrm{ATI},x}$ [N]	$F_{\mathrm{sensor},x}$ [N]	$F_{\mathrm{ATI},y}$ [N]	$F_{\mathrm{sensor},y}$ [N]	Error	Crosstalk
10.0068	10.3111	0.0348	0.2984	3.04%	2.63%
20.0174	20.5290	0.0666	0.3907	2.56%	1.62%
30.0094	30.7975	0.1440	0.6305	2.63%	1.62%
40.0048	40.8491	0.1724	0.2394	2.11%	0.17%
50.0008	50.8072	0.2039	−1.0004	1.61%	1.59%
−10.0331	−9.8362	0.2509	0.9131	1.96%	6.60%
−20.0243	−19.6538	0.4729	1.7386	1.85%	6.32%
−30.0273	−30.0811	0.7231	2.1185	0.18%	4.65%
−39.9968	−39.3684	0.8708	2.8858	1.57%	5.04%
−50.0032	−49.5680	1.0070	3.5859	0.87%	5.16%

**Table A2:** Mean force input measurements on the force sensor and the ATI mini45 for forces in the range of ±50 N along the *y*-axis.

$F_{\mathrm{ATI},y}$ [N]	$F_{\mathrm{sensor},y}$ [N]	$F_{\mathrm{ATI},x}$ [N]	$F_{\mathrm{sensor},x}$ [N]	Error	Crosstalk
10.0009	10.2204	−0.1837	0.7648	2.20%	5.81%
20.0760	21.0121	−0.3518	1.2867	4.66%	4.66%
30.0376	30.9650	−0.5091	1.6408	3.09%	3.77%
40.0171	41.2125	−0.7220	1.9972	2.99%	3.19%
50.0058	51.1176	−0.9583	2.4090	2.22%	2.90%
−10.0272	−9.6566	0.0964	0.1112	3.70%	0.15%
−20.0387	−19.6878	0.2204	−0.0881	1.75%	0.66%
−30.0071	−28.9638	0.3893	0.2397	3.48%	0.50%
−40.0236	−38.9780	0.5060	0.1669	2.61%	0.85%
−50.0071	−49.0965	0.6453	−0.1301	1.82%	1.03%
